# Exploring the Feasibility and Initial Impact of an mHealth-Based Disease Management Program for Chronic Ischemic Heart Disease: Formative Study

**DOI:** 10.2196/56380

**Published:** 2024-08-22

**Authors:** Takahiro Miki, Junya Yamada, Shinpei Ishida, Daisuke Sakui, Masashi Kanai, Yuta Hagiwara

**Affiliations:** 1 PREVENT Inc Aichi Japan; 2 Institute of Transdisciplinary Sciences for Innovation Kanazawa University Kanazawa Japan

**Keywords:** mobile health, chronic ischemic heart disease, disease management program, mobile phone, behavior change

## Abstract

**Background:**

Ischemic heart disease (IHD) is a leading cause of morbidity and mortality worldwide, requiring innovative management strategies. Traditional disease management programs often struggle to maintain patient engagement and ensure long-term adherence to lifestyle modifications and treatment plans. Mobile health (mHealth) technologies have emerged as a promising approach to address these challenges by providing continuous, personalized support and monitoring. However, the reported use and effectiveness of mHealth in the management of chronic diseases, such as IHD, have not been fully explored.

**Objective:**

The primary aim of this study was to evaluate the feasibility and initial impact of an mHealth-based disease management program on coronary risk factors, specifically focusing on low-density lipoprotein cholesterol (LDL-C) levels, in individuals with chronic IHD. This formative study assessed changes in LDL-C and other metabolic health indicators over a 6-month period to determine the initial impact of the program on promoting cardiovascular health and lifestyle modification.

**Methods:**

This study was conducted using data from 266 individuals enrolled in an mHealth-based disease management program between December 2018 and October 2022. Eligibility was based on a documented history of IHD, with participants undergoing a comprehensive cardiac risk assessment before enrollment. The program included biweekly telephone sessions, health tracking via a smartphone app, and regular progress reports to physicians. The study measured change in LDL-C levels as the primary outcome, with secondary outcomes including body weight, triglyceride levels, and other metabolic health indicators. Statistical analysis used paired 2-tailed *t* tests and stratified analyses to assess the impact of the program.

**Results:**

Participants experienced a significant reduction in LDL-C, with LDL-C levels decreasing from a mean of 98.82 (SD 40.92) mg/dL to 86.62 (SD 39.86) mg/dL (*P*<.001). The intervention was particularly effective in individuals with high baseline LDL-C levels. Additional improvements were seen in body weight and triglyceride levels, suggesting a broader impact on metabolic health. Program adherence and engagement metrics suggested high participant satisfaction and compliance.

**Conclusions:**

The results of this study suggest that the mHealth-based disease management program is feasible and has an initial positive impact on reducing LDL-C levels and improving metabolic health in individuals with chronic IHD. However, the study design does not allow for a definitive conclusion regarding whether mHealth-based disease management programs are more effective than traditional face-to-face care. Future studies are needed to further validate these findings and to examine the comparative effectiveness of these interventions in more detail.

## Introduction

### Background

Cardiovascular diseases, including ischemic heart disease (IHD), present distinct challenges and critical outcomes, setting them apart from other chronic conditions [1]. Effective management of IHD requires a highly specialized approach, especially in monitoring and controlling key health indicators such as low-density lipoprotein cholesterol (LDL-C) levels. Recognized as a crucial marker for heart disease risk, managing LDL-C is vital for halting IHD’s progression [2]. This management strategy encompasses both medical interventions and comprehensive lifestyle changes tailored to each patient’s unique health profile and risk factors. Essential lifestyle modifications include diet alteration, regular exercise, and smoking cessation [3]. However, implementing and maintaining these changes can be challenging for patients without ongoing support and motivation.

The application of mobile devices, their components, and related technologies to health care is known as mobile health (mHealth). Due to its significant potential to facilitate everyday behavior change and promote healthy lifestyles, mHealth is gaining worldwide popularity [4]. Recently, mHealth technologies have significantly advanced the management of chronic diseases, including IHD [5-8]. Recent studies and meta-analyses have shown the effectiveness of internet-based interventions for cardiovascular diseases that are adaptable to mobile devices and enhance patient monitoring and self-care education [9,10]. A study in Australia showed that web-based cognitive behavioral therapy improved psychological symptoms, medication adherence, and health behaviors in patients at a high risk of cardiovascular diseases [11]. Another study highlighted a mobile and web-based self-management program that significantly reduced hemoglobin A1c (HbA_1c_) levels in patients with diabetes [12]. This study demonstrates the potential of integrated digital platforms in chronic disease management. Further, a recent systematic review found mHealth apps effective in promoting lifestyle changes such as weight loss and exercise adherence in patients with chronic disease, emphasizing technology’s potential in treatment support [13]. A latest study for mHealth cardiac rehabilitation has demonstrated significant improvements in inner strength and resilience among older patients after myocardial infarction discharge. Through biweekly web-based training and support, the intervention group showed notable enhancements in these areas, contrasting with the control group’s stable scores. This indicates mHealth’s potential to boost self-care and quality of life in older patients after myocardial infarction [14]. These tools represent a new paradigm in health interventions, offering continuous, real-time monitoring and tailored health care delivery. mHealth is revolutionizing long-term illness management by providing current information and personalized care and enhancing patient engagement in their health journey. Now a vital component of health care, mHealth is particularly crucial in managing various chronic conditions. Its role in monitoring health and ensuring adherence to treatment plans is key to successful outcomes. The increasing cases of IHD and the availability of mHealth tools present new opportunities for effective disease management [15].

Previous studies on mHealth interventions for heart failure and IHD have primarily focused on LDL-C in the acute phase, with few exploring it as the primary outcome in the maintenance phase of IHD [16]. This lack of focus leaves a significant gap in understanding mHealth’s long-term efficacy in managing LDL-C levels in patients with IHD, where maintaining health improvements is often more challenging than initial success. Moreover, the effectiveness of mHealth goes beyond the technology itself; successful outcomes often depend on regular professional feedback and guidance [5,17,18].

The Mystar program, a Japanese mHealth-based disease management initiative, has undergone a 6-month trial led by medical professionals. It demonstrated notable improvements in blood pressure and weight for patients with various lifestyle diseases [19]. However, the lack of studies on the use of mHealth for the management of chronic diseases, such as IHD, requires focused studies on its feasibility and initial impacts.

### Aim

This study aimed to assess the feasibility and acceptability of an mHealth-based disease management program. A second aim was to assess the initial impact of the mHealth-based disease management program on individuals with IHD. It is expected to provide early evidence on how mHealth can support the ongoing management of IHD and potentially influence future chronic disease management strategies.

## Methods

### Study Setting

This study was designed to evaluate the impact of the Mystar program on LDL-C levels in patients with IHD using data from PREVENT Inc in Nagoya, Japan. The participant pool, drawn from employees or their dependents affiliated with health insurance associations, engaged in the Mystar program, a 6-month initiative focused on lifestyle modification managed by PREVENT Inc, from December 2018 to October 2022. Enrollment criteria hinged on a documented history of IHD. To ensure safety, participants with IHD underwent mandatory cardiac risk assessments before entering the program. These evaluations included (1) resting 12-lead electrocardiogram (ECG), (2) exercise ECG, and (3) echocardiography examination.

On the basis of these evaluations, the following exclusion criteria were applied specifically for patients with IHD: (1) participants with a Lown classification of 4b or higher on the resting ECG were excluded; (2) those showing positive ischemic responses on the exercise ECG were excluded, unless there was explicit consent from the attending physician for non–exercise-based interventions; and (3) participants with an ejection fraction of <45%, or moderate to severe valvular disease identified in the echocardiography examination, were also excluded.

In addition, the program excluded individuals who had been hospitalized for a primary diagnosis of heart failure or who were taking cardiotonic medications. Eligibility for patients with IHD was contingent upon clearance from these rigorous evaluations and explicit consent from their treating physicians. Participants who did not complete the 6-month course were excluded. Informed consent was obtained from all participants at baseline, with the understanding that data collected through the app may be used in future research efforts.

### Ethical Considerations

The study was conducted in compliance with the Declaration of Helsinki and approved by the Konan Women’s University Research Ethics Committee (approval number 2021008), ensuring adherence to human participant research ethics. Informed consent was obtained from all participants, with the data anonymized and deidentified to protect privacy and confidentiality. No financial compensation was provided to participants, who were free to withdraw at any time. All data presented were anonymized, and there are no images in the manuscript or multimedia appendix that could identify individual participants.

### mHealth-Based Disease Management Program （Mystar Program）

#### Overview of the Program Procedure

The Mystar program was developed as a mobile app compatible with both Android (Google LLC) and iOS (Apple Inc) platforms, designed to support individuals with various lifestyle-related conditions. The program was established through partnerships between PREVENT Inc and health insurance associations, with participants voluntarily enrolling from these associations.

#### Technology and Training

Participants begin by downloading the Mystar app to their smartphones and using it with wearable devices such as the Fitbit Alta HR (Fitbit Inc), the Fitbit Inspire HR (Fitbit Inc), and a GENEN Monitor (Kono ME Laboratory, Inc). This setup allows them to monitor health metrics such as body weight, physical activity, heart rate, and sleep patterns. No prior training is required. They receive instructions on how to use the devices and the app at the beginning of the program and learn through use under the guidance of dedicated coordinators who remain constant throughout the 6-month program.

#### Program Delivery

**Figure 1:**
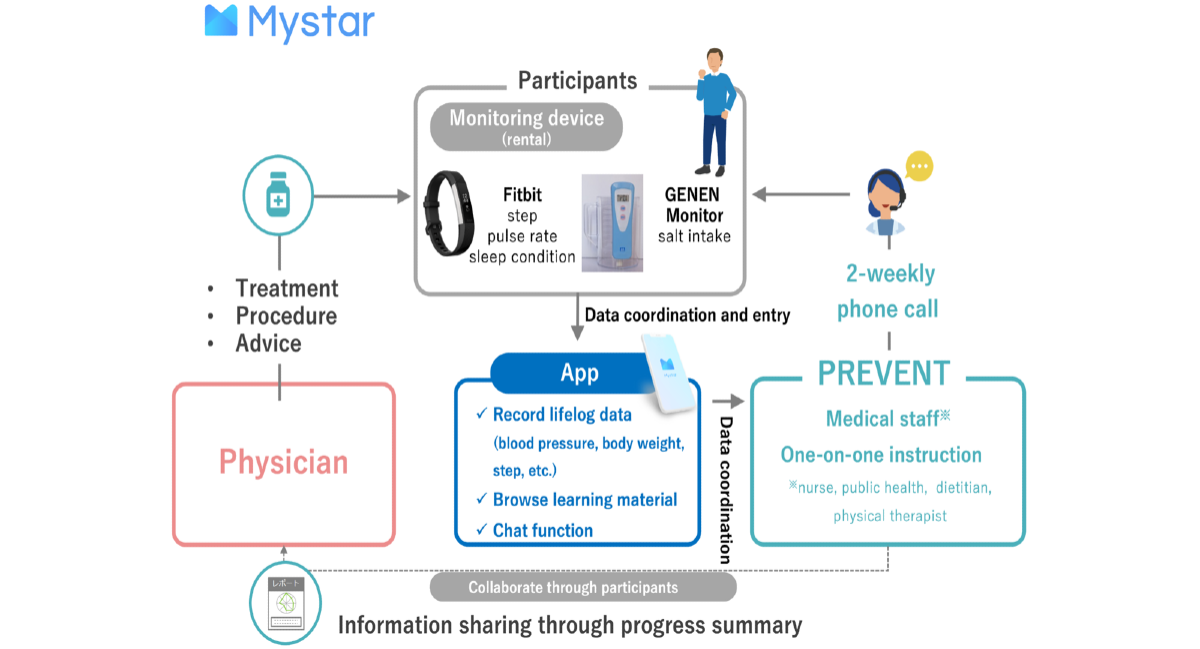
Overall diagram of the Mystar program.

The program is administered by a multidisciplinary health care team that includes nurses, dietitians, and physical therapists. Spanning approximately 6 months, it comprises 12 sessions with biweekly telephonic check-ins scheduled at convenient times for the participants. Each session is designed to identify and modify lifestyle habits to improve disease management, with comprehensive discussions on diet, exercise, and stress management. The initial phone session provides training on app use, followed by continuous assessment and adaptation of lifestyle habits. The intervention integrates educational content, personalized feedback, and behavior change support, using data from wearable devices for customization. Program goals are collaboratively set by participants and the medical team, focusing on long-term health outcomes and behavioral objectives for each session. Educational materials and tips for improving lifestyle habits are consistently provided, supplemented by motivational interviewing techniques and behavior change strategies. Moreover, for safety considerations, the program maintains close coordination with the participant’s primary care physician, particularly concerning the management of lifestyle disease scores and medication adjustments (Figure 1).

#### Data Security

Regarding data security, the program has obtained the JAPHIC mark, which complies with Japan’s personal information protection laws, and obtained information security management system (ISO27001), an international standard for information security. Data are stored on cloud servers with no external access allowed, and both 2-factor authentication and virtual private network connections are mandatory.

#### Program Evaluation

Upon the completion of the program, participants evaluate their satisfaction and change in their own behavior. Feedback is also provided through a survey that includes both multiple-choice and open-ended questions. This feedback focuses on several aspects of the program, including the overall experience, the effectiveness of the interventions, and areas for improvement. The insights gained from the survey are used to refine the program and ensure that it continues to evolve to better meet the needs of its users.

#### Data Collection

Data collected included personality traits, age, sex, BMI, physical activity level, systolic blood pressure, diastolic blood pressure, LDL-C, high-density lipoprotein cholesterol (HDL-C), fasting blood glucose, salt intake, and HbA_1c_ levels.

##### Sources of Data

Data were obtained from several sources: recent medical examinations, blood tests, data entered into the app by participants, and telephonic records from medical staff. Lifelog data encompassed morning home blood pressure readings, cholesterol levels, triglycerides, HbA_1c_, body weight, step count, and daily salt intake.

##### Collection Intervals

Lifelog data included body weight, blood pressure, and physical activity. Data used for analysis were the average values recorded in the app. These averages were calculated for the first week following the initial consultation (“pre”) and for the last week before the final consultation (“post”).

With regard to blood tests, measurements for LDL-C and other blood parameters were collected within specific time frames: from 365 days before to 30 days after the initial consultation for pretest results and from 30 days before to 365 days after the final consultation for posttest results. These periods were selected to accurately reflect the impact of the intervention on LDL-C levels around key study milestones.

##### Specific Measurement Methods

Blood pressure was measured using participants’ own devices, preferably oscillometric upper arm cuff monitors, in accordance with hypertension guidelines [20]. Conditions required a quiet environment with a comfortable room temperature; participants sitting in a chair with back support without crossing their legs; and no smoking, alcohol, or caffeine intake before measurement. The cuff was kept positioned at heart level. Measurements were taken in the early morning within 1 hour of awakening and before bedtime and recorded daily in the app.

Physical activity was monitored using either Fitbit Inspire2 or Inspire HR devices, which were provided at the start of the program, or participants’ own activity trackers. Participants were instructed to wear these devices continuously throughout the day. Data collected were automatically synced to the app. In cases where automatic synchronization failed, participants were asked to manually enter the previous day’s activity data the following day. The reliability of these devices has been confirmed in several studies [21].

### Outcomes

The primary outcome was the level of LDL-C, a critical indicator in the management of IHD. The secondary outcomes were body weight, HDL-C triglycerides, HbA_1c_, uric acid, blood pressure, sleep duration per day, and daily step count.

### Statistical Analysis

Descriptive statistics on participant demographics and survey results were compiled. Missing data were addressed using the hot deck imputation technique. Continuous variables were presented as means with SDs, and categorical variables were described using counts and percentages. The median and IQR were used to report the duration of app use. The primary method to assess the effectiveness of the Mystar program was paired 2-tailed *t* tests, which compared the baseline data (mean of the 2 weeks following the initial phone session) to the postintervention data (mean of the 2 weeks preceding the final phone session). In addition, we conducted a stratified analysis by dividing participants into 2 groups based on their initial LDL-C levels. We used 100 mg/dL as the threshold for the LDL-C management target in these guidelines [22]. This analysis allowed us to evaluate changes in key health indicators, such as LDL-C, body weight, HDL-C, triglycerides, HbA_1c_, uric acid, morning systolic blood pressure, salt intake, and sleep duration, both before and after the intervention within each group. To further examine the program’s impact, we performed a comparative analysis between these independent groups. This involved assessing the average changes in various health indicators in participants with initial LDL-C levels ≥100 mg/dL and those with levels <100 mg/dL. This comparison aimed to elucidate the differential effects of the intervention across these distinct groups, and the results included the 95% CIs computed for these differences. The analysis yielded coefficients, SEs, *t* values, *P* values, and 95% CIs for each personality trait. The threshold for statistical significance was set at *P*<.05. For these computations, we used the R software (version 4.0.2; R Foundation for Statistical Computing).

## Results

### The Characteristics of the Participants and Results of the Participant Survey

The study initially enrolled 270 participants; after the exclusion of 4 participants, the final number of participants was 266, resulting in a retention rate of 98.5%. Reasons for dropout included cancer diagnosis, inability to continue due to retirement, and personal reasons for 2 participants. There were no withdrawals due to serious events, such as death or relapse. Participant characteristics are shown in Table 1. In addition, no adverse events, defined as any adverse medical event, including cardiovascular death or new vascular events requiring hospitalization, occurred during the program. The results of the participant survey about the program are shown in Table 2. The response rate was 22.2%, with 59 of the 266 participants responding. In addition, 25 (9.3%) of these respondents provided free-text comments. The free-text comments are included in Multimedia Appendix 1.

**Table 1:** Participant demographics and baseline characteristics.

Characteristics			All (n=266)		Group with initial LDL-C^a^ values ≥100 mg/dL (n=129)		Group with initial LDL-C values <100 mg/dL (n=137)
Sex (male), n (%)			247 (92.86)		115 (89.15)		132 (96.35)
Age (y), mean (SD)			56.39 (6.02)		56.21 (6.46)		56.57 (5.59)
Hypertension, n (%)			197 (74.06)		93 (72.09)		104 (75.91)
Diabetes mellitus, n (%)			135 (50.75)		70 (54.26)		65 (47.45)
Dyslipidemia, n (%)			182 (68.42)		82 (63.57)		100 (72.99)
Cerebrovascular accident, n (%)			22 (8.27)		11 (8.52)		11 (8.03)
Ischemic myocardial infarction, n (%)			112 (43.12)		41 (31.78)		71 (51.82)
Medical use, n (%)			173 (65.04)		73 (56.59)		100 (72.99)
Height (cm), mean (SD)			162.60 (26.61)		165.98 (16.79)		159.42 (33.06)
Weight (kg), mean (SD)			77.51 (17.53)		79.23 (18.82)		75.89 (16.13)
BMI (kg/m²), mean (SD)			26.14 (6.88)		27.49 (5.78)		24.86 (7.59)
LDL-C (mg/dL), mean (SD)			98.82 (40.92)		131.56 (26.19)		68.00 (25.36)
HDL-C^b^ (mg/dL), mean (SD)			47.88 (21.46)		51.27 (14.05)		44.68 (26.28)
Triglycerides (mg/dL), mean (SD)			145.83 (98.52)		166.09 (95.88)		126.76 (97.48)
HbA_1c_^c^ (%), mean (SD)			5.69 (2.08)		6.17 (1.89)		5.24 (2.16)
Uric acid level, (mg/dL) mean (SD)			5.13 (1.86)		5.63 (1.78)		4.66 (1.82)
SBP^d^, (mm Hg) mean (SD)			114.78 (33.85)		113.88 (36.36)		115.62 (31.42)
DBP^e^ (mm Hg), mean (SD)			73.58 (22.65)		74.55 (23.50)		72.67 (21.87)
Estimated salt intake (g/day), mean (SD)			8.79 (3.82)		8.68 (3.71)		8.90 (3.94)
Sleep duration (hours), mean (SD)			5.50 (1.39)		5.39 (1.48)		5.61 (1.31)
Step count (steps/day), mean (SD)			8564.95 (3948.80)		8092.48 (3776.04)		9009.82 (4068.49)
**Personal traits, mean (SD)**							
	Neuroticism	7.85 (2.13)		7.95 (2.01)		7.76 (2.24)	
	Extraversion	8.61 (2.55)		8.68 (2.47)		8.53 (2.63)	
	Openness	8.76 (2.28)		8.81 (2.36)		8.70 (2.20)	
	Agreeableness	10.08 (1.85)		9.99 (1.95)		10.16 (1.77)	
	Conscientiousness	8.13 (2.48)		7.94 (2.37)		8.31 (2.58)	

^a^LDL-C: low-density lipoprotein cholesterol.

^b^HDL-C: high-density lipoprotein cholesterol.

^c^HbA_1c_: hemoglobin A1c.

^d^SBP: systolic blood pressure.

^e^DBP: diastolic blood pressure.

**Table 2:** Results of the participant survey about the program^a^.

Item		Scores, mean (SD)
**User satisfaction with the mobile health app (1-5)**		
	1. Integration of the app and external devices	3.72 (1.25)
	2. Ease of app operation	4.15 (1.11)
	3. Lifelog recording	4.10 (1.16)
	4. Diet recording	3.89 (1.25)
	5. Reading material	4.00 (1.16)
	6. Chat function	4.33 (1.13)
	7. Satisfaction with the assigned coordinator	4.77 (0.96)
	8. Appointment scheduling method	4.59 (1.04)
	9. Advice from the consultation coordinator	4.72 (1.00)
	10. Goal setting	4.54 (1.06)
	11. Content of progress reports	4.47 (1.07)
	12. Interactions via chat	4.57 (1.07)
**Impact on personal behavior change (1-5)**		
	1. Change in lifestyle habits after program completion	4.42 (1.06)
	2. Formation of good habits	4.49 (1.01)
	3. Improvement of bad habits	4.05 (1.15)
	4. Active engagement with the program	4.32 (1.07)
	5. Perceived improvement in physical health	3.39 (1.09)

^a^The response rate was 22.2%, with responses received from 59 of the 266 participants on a scale from 1 to 5, where 1 is the lowest and 5 is the highest.

### Preintervention and Postintervention Health Metric Analysis

Table 3 shows the impact of the program on health indicators, with notable improvements after the intervention. Key findings include a significant reduction in LDL-C from a mean of 98.82 (SD 40.92) to 86.62 (SD 39.86) mg/dL and a significant reduction in body weight from 77.51 (SD 17.53) kg to 72.39 (SD 19.84) kg. Although HDL-C levels remained stable, triglycerides decreased significantly, reinforcing the lipid management benefits of the program. The slight decrease in HbA_1c_, although not statistically significant, indicates a positive trend in glycemic control. Other health measures, such as uric acid and morning systolic blood pressure, did not show substantial changes, but overall trends suggest an improvement in cardiovascular health. Participants also showed increased physical activity, as evidenced by an increase in daily steps from 8564.95 (SD 3948.80) to 9309.91 (SD 4440.22), along with a trend toward reduced salt intake and stable sleep duration.

**Table 3:** Preintervention and postintervention health metric comparison^a^.

Indicator	Preintervention metric, mean (SD)	Postintervention metric, mean (SD; 95% CI)	*P* value
Body weight	77.51 (17.53)	72.39 (19.84; –8.31 to –1.94)	<.001
LDL-C^b^ (mg/dL)	98.82 (40.92)	86.62 (39.86; –19.06 to –5.33)	<.001
HDL-C^c^ (mg/dL)	47.88 (21.46)	47.62 (20.59; –3.83 to 3.32)	.86
Triglycerides (mg/dL)	145.83 (98.52)	115.89 (77.12; –44.98 to –14.91)	<.001
HbA_1c_ (%)^d^	5.69 (2.08)	5.46 (1.87; –0.56 to 0.11)	.13
Uric acid (mg/dL)	5.13 (1.86)	5.08 (1.93; –0.38 to 0.26)	.71
SBP (mm Hg)^e^	114.78 (33.85)	112.77 (35.21; –7.88 to 3.86)	.41
DBP (mm Hg)^f^	73.58 (22.65)	73.41 (21.52; –3.93 to 3.58)	.92
Salt intake (g/day)	8.79 (3.82)	8.32 (3.65; –1.11 to 0.16)	.09
Sleep duration (hours)	5.50 (1.39)	5.36 (1.55; –0.39 to 0.11)	.13
Steps per day	8564.95 (3948.80)	9309.91 (4440.22; 30.87 to 1459.06)	.01

^a^This table provides a detailed comparison of health metrics before and after the 6-month intervention using the Mystar mHealth-based disease management program in patients with ischemic heart disease. Conducted in Nagoya, Japan, from December 2018 to October 2022, the study involved 266 participants and assessed changes in body weight, blood pressure (systolic and diastolic), lipid profiles (low-density lipoprotein, high-density lipoprotein, and triglycerides), hemoglobin A1c, uric acid levels, salt intake, sleep duration, and daily steps. The table shows mean values with SDs for each metric before and after the intervention, along with *P* values and 95% CIs to illustrate the program’s impact on these health outcomes.

^b^LDL-C: low-density lipoprotein cholesterol.

^c^HDL-C: high-density lipoprotein cholesterol.

^d^HbA_1c_: hemoglobin A1c.

^e^SBP: systolic blood pressure.

^f^DBP: diastolic blood pressure.

### Comparison of Health Indicators in Participants With Initial LDL-C Levels ≥100 mg/dL

In a group of 129 participants with initial LDL-C levels ≥100 mg/dL, significant health improvements were observed following an intervention (*P*<.001). The analysis revealed a substantial reduction in LDL-C levels, from a mean of 131.56 (SD 26.19) to 96.17 (SD 40.66) mg/dL, with a *P* value of <.001. Body weight also decreased notably, from 79.23 (SD 18.82) to 72.23 (SD 21.08) kg, indicating effective weight management. However, HDL-C levels showed a slight decrease without statistical significance, from 51.27 (SD 14.05) to 48.29 (SD 18.63) mg/dL. Triglyceride levels significantly dropped from 166.09 (SD 95.88) to 130.05 (SD 75.39) mg/dL. HbA_1c_ levels improved from 6.17% (SD 1.89%) to 5.48% (SD 1.93%), and uric acid levels also decreased from 5.63 (SD 1.78) to 5.16 (SD 1.95) mg/dL. Morning systolic blood pressure showed a reduction, although not statistically significant, from 113.88 (SD 36.36) to 107.58 (SD 41.59) mm Hg. Morning diastolic blood pressure slightly decreased from 74.55 (SD 23.50) to 71.99 (SD 23.12), and salt intake was reduced from 8.68 (SD 3.71) to 7.72 (SD 3.45) g/day. Sleep duration showed a minor decrease from 5.39 (SD 1.48) to 5.19 (SD 1.70) hours. The details were described in Table 4.

**Table 4:** Preintervention and postintervention health metrics for participants with initial low-density lipoprotein cholesterol (LDL-C) levels ≥100 mg/dL^a^.

Indicator	Preintervention metric, mean (SD)	Postintervention metric, mean (SD; 95% CI)	*P* value
Body weight (kg)	79.23 (18.82)	72.23 (21.08; –11.87 to –2.12)	<.001
LDL-C (mg/dL)	131.56 (26.19)	96.17 (40.66; –43.73 to –27.04)	<.001
HDL-C^b^ (mg/dL)	51.27 (14.05)	48.29 (18.63; –7.01 to 1.05)	.09
Triglycerides (mg/dL)	166.09 (95.88)	130.05 (75.39; –57.09 to –15.00)	<.001
HbA_1c_^c^ (%)	6.17 (1.89)	5.48 (1.93; –1.16 to –0.23)	<.001
Uric acid (mg/dL)	5.63 (1.78)	5.16 (1.95; –0.93 to –0.02)	.03
SBP^d^ (mm Hg)	113.88 (36.36)	107.58 (41.59; –15.84 to 3.23)	.09
DBP^e^ (mm Hg)	74.55 (23.50)	71.99 (23.12; –8.24 to 3.13)	.31
Salt intake (g/day)	8.68 (3.71)	7.72 (3.45; –1.84 to –0.09)	.01
Sleep duration (hours)	5.39 (1.48)	5.19 (1.70; –0.59 to 0.19)	.18
Steps (steps/day)	8142 (3773.2)	8684 (4111.0; –428.1 to 1512.8)	.15

^a^This table shows health changes in 129 participants with initial low-density lipoprotein cholesterol (LDL-C) levels >100 mg/dL from a study at PREVENT Inc, Japan (2018-2022). It examines the effect of a mobile health–based disease management program on cardiovascular risk factors such as body weight, LDL-C, high-density lipoprotein, triglycerides, hemoglobin A1c (HbA_1c_), uric acid, blood pressure, salt intake, sleep, and steps. Significant changes in LDL-C, body weight, triglycerides, and HbA_1c_ were noted after the intervention, with *P* values and 95% CIs highlighting these outcomes.

^b^HDL-C: high-density lipoprotein cholesterol.

^c^HbA_1c_: hemoglobin A1c.

^d^SBP: systolic blood pressure.

^e^DBP: diastolic blood pressure.

### Comparison of Health Indicators in Participants With Initial LDL-C Levels <100 mg/dL

In a cohort of 137 participants with initial LDL-C levels <100 mg/dL, varied health outcomes were observed after the intervention (Table 5). LDL-C levels increased slightly but significantly, from a mean of 67.54 (SD 25.26) mg/dL to 77.13 (SD 37.01) mg/dL, with a *P* value of .01. Body weight showed a significant reduction, decreasing from 75.93 (SD 16.04) kg to 72.51 (SD 18.60) kg. HDL-C levels saw a nonsignificant increase from 44.36 (SD 26.28) mg/dL to 46.54 (SD 22.13) mg/dL. Triglyceride levels notably decreased from 126.93 (SD 97.98) mg/dL to 102.44 (SD 77.06) mg/dL. HbA1c levels showed a slight but nonsignificant increase from 5.22% (SD 2.15%) to 5.43% (SD 1.82%). Uric acid levels increased from 4.63 (SD 1.82) mg/dL to 4.97 (SD 1.91) mg/dL, although not significantly. Morning systolic blood pressure slightly increased from 116.15 (SD 31.12) mm Hg to 117.83 (SD 27.33) mm Hg, and morning diastolic blood pressure decreased from 74.55 (SD 23.50) mm Hg to 71.99 (SD 23.12) mm Hg, both changes being statistically nonsignificant. Salt intake remained nearly constant, changing from 8.92 (SD 3.95) g/day to 8.90 (SD 3.78) g/day, and sleep duration slightly decreased from 5.60 (SD 1.31) hours to 5.53 (SD 1.40) hours.

**Table 5:** Health outcomes after the intervention in participants with initial low-density lipoprotein cholesterol (LDL-C) levels <100 mg/dL^a^.

Indicator	Preintervention indicator, mean (SD)	Postintervention indicator, mean (SD; 95% CI)	*P* value
Body weight (kg)	75.93 (16.04)	72.51 (18.60; –7.56 to 0.72)	.01
LDL-C (mg/dL)	67.54 (25.26)	77.13 (37.01; 2.03 to 17.15)	.01
HDL-C^b^ (mg/dL)	44.36 (26.28)	46.54 (22.13; –3.61 to 7.98)	.35
Triglycerides (mg/dL)	126.93 (97.98)	102.44 (77.06; –45.52 to –3.47)	.02
HbA_1c_^c^ (%)	5.22 (2.15)	5.43 (1.82; –0.26 to 0.69)	.37
Uric acid (mg/dL)	4.63 (1.82)	4.97 (1.91; –0.11 to 0.78)	.13
SBP^d^ (mm Hg)	116.15 (31.12)	117.83 (27.33; –5.30 to 8.67)	.59
DBP^e^ (mm Hg)	74.55 (23.50)	71.99 (23.12; –8.24 to 3.13)	.31
Salt intake (g/day)	8.92 (3.95)	8.90 (3.78; –0.94 to 0.90)	.96
Sleep duration (hours)	5.60 (1.31)	5.53 (1.40; –0.39 to 0.25)	.53
Steps (steps/day)	9009.8 (4068.5)	9986.2 (4606.5; –52.8 to 2005.5)	.01

^a^This table shows health changes in 137 participants with initial low-density lipoprotein cholesterol (LDL-C) <100 mg/dL from a study at PREVENT Inc, Japan (2018-2022). It tracks changes in body weight, lipid profiles (LDL-C, high-density lipoprotein cholesterol [HDL-C], and triglycerides), hemoglobin A1c (HbA_1c_), uric acid, blood pressure, salt intake, sleep, and steps after the mHealth-based disease management program. Key results include a significant increase in LDL-C and decreases in body weight and triglycerides. Other measures such as HDL-C, HbA_1c_, and blood pressure changed but not significantly. The table lists mean values, SDs, *P* values, and 95% CIs, showing the program’s impact on these patients.

^b^HDL-C: high-density lipoprotein cholesterol.

^c^HbA_1c_: hemoglobin A1c.

^d^SBP: systolic blood pressure.

^e^DBP: diastolic blood pressure.

### Comparison of Changes in Health Indicators Between the Group With Initial LDL-C Levels ≥100 mg/dL and the Group With Initial LDL-C Levels <100 mg/dL

Table 6 presents a comparative analysis of health indicator changes between the group with initial LDL-C levels ≥100 mg/dL and the group with initial LDL-C levels <100 mg/dL. In the group with LDL-C ≥100 mg/dL, LDL-C decreased significantly by an average of 35.39 (SD 45.06) mg/dL. In contrast, the group with LDL-C <100 mg/dL saw an average increase of 9.64 (SD 44.18) mg/dL, highlighting a notable disparity (–45.02) between the groups. Other health parameters such as body weight, HDL-C, triglycerides, HbA_1c_, uric acid, and blood pressure also varied between these groups, underlining the differential impact of the intervention based on initial LDL-C levels. This analysis underscores the tailored effectiveness of the mHealth-based disease management program, particularly for individuals with initially higher LDL-C levels.

**Table 6:** Comparative analysis of health indicator changes by initial low-density lipoprotein cholesterol (LDL-C) levels^a^.

Indicator	Group with initial LDL-C values ≥100 mg/dL (SD)	Group with initial LDL-C values <100 mg/dL (SD)	Difference in changes (95% CI)	*P* value
Body weight (kg)	–7.00 (19.86)	–3.36 (15.23)	–3.64 (–7.77 to 0.50)	.09
LDL-C (mg/dL)	–35.39 (45.06)	9.64 (44.18)	–45.02 (–55.76 to –34.29)	<.001
HDL-C^b^ (mg/dL)	–2.98 (19.98)	2.30 (26.82)	–5.28 (–10.94 to 0.38)	.07
Triglycerides (mg/dL)	–36.05 (102.82)	–24.20 (114.65)	–11.85 (–37.99 to 14.29)	.38
HbA_1c_^c^ (%)	–0.69 (2.19)	0.21 (2.66)	–0.90 (–1.48 to –0.32)	.01
Uric acid (mg/dL)	–0.48 (2.48)	0.34 (2.51)	–0.82 (–1.42 to –0.22)	.01
SBP^d^ (mm Hg)	–6.30 (42.46)	2.03 (36.11)	–8.34 (–17.84 to 1.16)	.09
DBP^e^ (mm Hg)	–2.56 (28.22)	2.07 (27.24)	–4.63 (–11.30 to 2.04)	.18
Salt intake (g/day)	–0.97 (4.07)	–0.01 (4.88)	–0.96 (–2.04 to 0.12)	.08
Sleep duration (hours)	–0.20 (1.66)	–0.08 (1.29)	–0.12 (–0.48 to 0.24)	.52
Steps (steps/day)	542.3 (4116.54)	976.4 (3 781.34)	–434.0 (–1392.1 to 524.1)	.33

^a^This table compares health indicator changes between the group with initial LDL-C levels ≥100 mg/DL and the group with initial LDL-C levels <100 mg/DL from a retrospective study at PREVENT Inc, Japan (2018-2022). It shows significant LDL-C reduction in the higher LDL-C group, contrasting with an increase in the lower LDL-C group, indicating the intervention’s differential impact. Changes in body weight, high-density lipoprotein, triglycerides, hemoglobin A1c, uric acid, and blood pressure were also analyzed, highlighting how initial LDL-C levels influence the program’s effectiveness. This table provides the differences in changes, *P* values, and 95% CIs, illustrating the program’s tailored impact on cardiovascular risk factors.

^b^HDL-C: high-density lipoprotein cholesterol.

^c^HbA_1c_: hemoglobin A1c.

^d^SBP: systolic blood pressure.

^e^DBP: diastolic blood pressure.

## Discussion

### Principal Findings

This study assessed the feasibility and initial impact of an mHealth-based disease management program on coronary risk factors, specifically focusing on LDL-C levels, in individuals with chronic IHD. The program was well organized. There was low attrition and high satisfaction. The primary finding revealed a substantial decrease in LDL-C, from an average of 98.82 (SD 40.92) to 86.62 (SD 39.86) mg/dL, highlighting the program’s effectiveness in cardiovascular risk management. Individuals with initially high LDL-C levels exhibited the most pronounced benefits, suggesting the program’s potential for targeted risk reduction in higher-risk groups.

The guidelines for reporting of health interventions using mobile phones have been established, providing a basis for assessing the reliability and challenges of mHealth interventions in a structured manner [23]. These considerations allow for a preliminary assessment of the program’s reliability and the challenges it faces at this stage. The successful implementation of the Mystar program depends on a region’s technological infrastructure, which is vital for deploying mHealth interventions effectively. Japan boasts a substantial mobile penetration rate, with > 90% of the population owning smartphones and having access to 4G LTE networks. This high level of connectivity indicates that mHealth interventions can be readily adopted in Japan, presenting minimal barriers to entry for its population. Since its inception in 2016, the Mystar program has evolved significantly, with several years now under its current app-based format. To date, approximately 8500 individuals have participated through collaborations with 150 health insurance associations, and in recent years, agreements with local municipalities have been established. Consistently low dropout rates and high satisfaction levels similar to those observed in this study have been maintained. A key factor contributing to these outcomes is the program’s interactive nature, where coaches engage regularly with participants via chat and phone calls to provide feedback. Research suggests that the effectiveness of mHealth programs is significantly enhanced by such interactive elements, underscoring the importance not merely of using digital tools but also of integrating timely and appropriate feedback from medical professionals and other health experts. This approach helps maximize both engagement and outcomes [24].

However, the use of mHealth within Japan’s insurance system is still relatively novel, with only a few examples to date [25,26]. Currently, our program primarily operates through contracts with health insurance associations, but expanding access to include coverage under the national insurance system could be a viable strategy to reach a broader audience. Participants in this study were subject to stringent inclusion criteria, including confirmed diagnoses and the ability to coordinate with physicians. As we consider expanding the reach of this program, options such as making it accessible to individuals without formal diagnoses might be explored. However, this approach also introduces potential risks that must be carefully weighed. Moving forward, it is crucial to balance broadening access with maintaining the integrity and effectiveness of the program.

Although participants were not involved in the development of this app, we received feedback on its use through a questionnaire at the end of the program. The overall satisfaction with the mobile app was generally positive. Participants rated the ease of app operation highly (average 4.15), indicating that the user interface was well designed and user-friendly. Similarly, the lifelog recording and diet recording functions were rated favorably (averages of 4.10 and 3.89, respectively), suggesting that these features effectively met the needs of users. However, the slightly lower score for diet recording indicates room for improvement in this feature. Communication features such as the chat function and interactions with health care coordinators received high ratings (averages of 4.33 and 4.77, respectively). These scores reflect the program’s success in facilitating effective communication between patients and health care providers, which is crucial for the management of chronic conditions such as IHD. The mHealth program is effective in being an interactive program [24]. Regarding behavior change, the program’s impact on behavior change was also positive, with participants reporting significant improvements in lifestyle habits (average 4.42). The high scores for the formation of good habits (average 4.49) and active engagement with the program (average 4.32) highlight the effectiveness of the mHealth intervention in motivating participants to adopt healthier lifestyles. Despite the overall positive feedback, the lowest score in the survey was for perceived improvement in physical health (average 3.39). This suggests that while participants recognized the benefits of the program in terms of lifestyle changes, these changes did not necessarily translate into a significant improvement in perceived physical health. This discrepancy underscores the need for the ongoing evaluation of how lifestyle changes through mHealth interventions impact long-term health outcomes.

In contrast, there are also areas that need improvement. While the dropout rate for the program was exceptionally low at 98.5% (266/270), it is important to consider that this high retention may be partially attributed to the voluntary nature of participation. As participation was optional, it is likely that those who engaged were already somewhat motivated to make health-related changes. This self-selection bias might limit the program’s reach, particularly among less motivated subgroups, such as those who are indifferent or resistant to behavior change. Although these individuals are often the ones who could benefit the most from such interventions, they are also the least likely to participate under voluntary conditions. In addition, the requirement for a physician’s diagnosis and permission to participate introduces another layer of complexity and a potential barrier to entry. While these measures are crucial for ensuring safety, they could restrict access to the program for a broader audience. Looking forward, exploring methods to lower these barriers, possibly by providing alternative pathways for engagement that do not compromise safety, could help make the program more inclusive and accessible to a wider population, irrespective of their current stage of behavior change or medical diagnosis. Moreover, the program’s availability is currently confined to a single company responsible for both development and administration. This exclusivity limits the ability of other organizations to implement the program. Furthermore, the requirement for specialized training for instructors restricts the pool of individuals who can provide guidance, posing challenges for the program’s reproducibility and standardization. As for app use, the absence of preliminary training means that user proficiency with mobile apps can vary significantly. This variability could complicate the setup process and, consequently, impact the intervention’s effectiveness. The program structure, which involves biweekly phone interventions and weekly chats conducted by the same individual over 6 months, exhibits a lack of personnel flexibility. This rigidity might hinder the scalability of the program, as expanding the service without a proportional increase in qualified staff could prove challenging. In conclusion, this study highlights several barriers to the widespread adoption of an mHealth program, including self-selection bias where highly motivated individuals are more likely to participate and stringent requirements such as physician approval that limit broader accessibility. Addressing these issues by lowering entry barriers and enhancing flexibility in program delivery could significantly expand the reach and effectiveness of the intervention to include a more diverse participant base.

Regarding the initial impact of this program, these results are consistent with previous research on mHealth interventions for IHD, where both app-based and SMS text messaging strategies have been shown to be effective in managing dyslipidemia and promoting healthier lifestyle habits [27]. Similar outcomes have been reported in pharmacist-led lipid management programs that used face-to-face, community-based approaches [28,29]. Our study’s focus on lifestyle changes, without specific medication directives, adds to the evidence supporting nonpharmacological strategies in cardiovascular risk management. The observed improvements in dietary habits, exercise, stress management, smoking cessation, and alcohol moderation corroborate the effectiveness of lifestyle interventions in enhancing cardiovascular health. The mHealth-based disease management program in this study emphasized lifestyle habit improvements. Such modifications, including significant LDL-C reduction in type 2 diabetes and other contexts such as yoga, demonstrate a broad impact on health [30]. Lifestyle modification may be crucial for reducing LDL-C and aiding in weight loss, and it has been shown to be effective not only in face-to-face interventions but also in mHealth apps [31]. Mobile apps, with their convenience and adaptability, encourage participation and lasting behavior change. This study’s biweekly phone-based interventions, accommodating participants’ schedules, likely contributed to high retention and success rates. For instance, a comprehensive mHealth program combining an app, activity tracker, and counseling showed significant improvements in obesity management [32]. Personalized mobile interventions, using data-driven customization, exhibited moderate positive effects on lifestyle behaviors [33]. This study found potential efficacy in personalized programs where medical professionals provide feedback. However, the research design used does not allow for a definitive conclusion regarding whether these programs are more effective than traditional, face-to-face treatments. Future study is necessary to further validate these findings and explore the comparative effectiveness of these intervention methods in greater detail.

### Limitations

This study has several limitations. First, the lack of a control group is a significant limitation. Without a comparison group, it is challenging to firmly establish the effectiveness of the intervention. The changes observed could be influenced by external factors unrelated to the intervention, such as seasonal variations in health behaviors or concurrent health initiatives in the community. Second, variability in the delivery of the intervention by different facilitators, despite having standardized protocols, introduces potential inconsistencies. While the program was guided by structured rules and a unified strategy, the individual approach of each facilitator might have affected the participants’ experience and outcomes. This variability could have led to differences in how participants received and perceived the intervention, thereby impacting its effectiveness. Third, the study primarily targeted working individuals, raising questions about the results’ applicability to other demographic groups. It remains unclear whether the observed benefits would extend to nonworking individuals, the older population, or those with different health conditions. Fourth, we could not get detailed information on how participants used medications such as statins. This is a big limitation because our study’s design and difficulty in getting full medical records made it hard to know about the participants’ medication use. Medications play a key role in managing IHD because they help control risk factors and improve the results of lifestyle changes. Including information on how participants used their medications would have given us a clearer picture of what contributed to the health outcomes we saw. It would have also helped us understand how medications and the mHealth program might work together to help people with IHD. Finally, the self-reported nature of some of the data, particularly regarding lifestyle changes and health behaviors, may introduce reporting bias. Participants’ perceptions and willingness to report certain behaviors accurately could have influenced the data quality, potentially leading to overestimations or underestimations of the intervention’s impact.

### Conclusions

In conclusion, the mHealth-based disease management program was well organized and achieved high levels of participant satisfaction. It shows promising potential for managing LDL-C and other cardiovascular risk factors. Future research should address the limitations of this study through randomized controlled trials and research designs that mitigate bias. In addition, it is important to focus on the broad applicability of such interventions across diverse populations and risk profiles. This will allow for the exploration of how mHealth technologies can support individual health management and tailor treatments to specific needs.

## Appendix

Multimedia Appendix 1 Free description of comments on the use of the program.

## Abbreviations

HbA_1c_: hemoglobin A_1c_
HDL-C: high-density lipoprotein cholesterol
IHD: ischemic heart disease
LDL-C: low-density lipoprotein cholesterol
mHealth: mobile health

## Data Availability

The data sets generated during and analyzed during this study are available from the corresponding author on reasonable request.

## References

[1] Noncommunicable diseases progress monitor 2022. World Health Organization.

[2] Baigent C, Blackwell L, Emberson J, Holland LE, Reith C, Bhala N, Peto R, Barnes EH, Keech A, Simes J, Collins R, Cholesterol Treatment Trialists’ (CTT) Collaboration (2010). Efficacy and safety of more intensive lowering of LDL cholesterol: a meta-analysis of data from 170,000 participants in 26 randomised trials. Lancet.

[3] Jain KK (2017). Personalized management of cardiovascular disorders. Med Princ Pract.

[4] Nahar P, Kannuri NK, Mikkilineni S, Murthy GV, Phillimore P (2017). mHealth and the management of chronic conditions in rural areas: a note of caution from southern India. Anthropol Med.

[5] Indraratna P, Tardo D, Yu J, Delbaere K, Brodie M, Lovell N, Ooi SY (2020). Mobile phone technologies in the management of ischemic heart disease, heart failure, and hypertension: systematic review and meta-analysis. JMIR Mhealth Uhealth.

[6] Koehler F, Winkler S, Schieber M, Sechtem U, Stangl K, Böhm M, Boll H, Baumann G, Honold M, Koehler K, Gelbrich G, Kirwan BA, Anker SD (2011). Impact of remote telemedical management on mortality and hospitalizations in ambulatory patients with chronic heart failure: the telemedical interventional monitoring in heart failure study. Circulation.

[7] Chen C, Li X, Sun L, Cao S, Kang Y, Hong L, Liang Y, You G, Zhang Q (2019). Post-discharge short message service improves short-term clinical outcome and self-care behaviour in chronic heart failure. ESC Heart Fail.

[8] Vuorinen AL, Leppänen J, Kaijanranta H, Kulju M, Heliö T, van Gils M, Lähteenmäki J (2014). Use of home telemonitoring to support multidisciplinary care of heart failure patients in Finland: randomized controlled trial. J Med Internet Res.

[9] Vernooij JW, Kaasjager HA, van der Graaf Y, Wierdsma J, Grandjean HM, Hovens MM, de Wit GA, Visseren FL (2012). Internet based vascular risk factor management for patients with clinically manifest vascular disease: randomised controlled trial. BMJ.

[10] Liu S, Dunford SD, Leung YW, Brooks D, Thomas SG, Eysenbach G, Nolan RP (2013). Reducing blood pressure with internet-based interventions: a meta-analysis. Can J Cardiol.

[11] Glozier N, Christensen H, Naismith S, Cockayne N, Donkin L, Neal B, Mackinnon A, Hickie I (2013). Internet-delivered cognitive behavioural therapy for adults with mild to moderate depression and high cardiovascular disease risks: a randomised attention-controlled trial. PLoS One.

[12] Quinn CC, Shardell MD, Terrin ML, Barr EA, Ballew SH, Gruber-Baldini AL (2011). Cluster-randomized trial of a mobile phone personalized behavioral intervention for blood glucose control. Diabetes Care.

[13] Debon R, Coleone JD, Bellei EA, De Marchi AC (2019). Mobile health applications for chronic diseases: a systematic review of features for lifestyle improvement. Diabetes Metab Syndr.

[14] Salarvand S, Farzanpour F, Gharaei HA (2024). The effect of personalized mobile health (mHealth) in cardiac rehabilitation for discharged elderly patients after acute myocardial infarction on their inner strength and resilience. BMC Cardiovasc Disord.

[15] Cruz-Ramos NA, Alor-Hernández G, Colombo-Mendoza LO, Sánchez-Cervantes JL, Rodríguez-Mazahua L, Guarneros-Nolasco LR (2022). mHealth apps for self-management of cardiovascular diseases: a scoping review. Healthcare (Basel).

[16] Megat Kamaruddin PS, Mohammed Nawi A, Abdul Manaf MR, Yaman MN, Abd Malek AM (2023). A meta-analysis of eHealth interventions on ischaemic heart disease health outcomes. Glob Heart.

[17] DiClemente CC, Marinilli AS, Singh M, Bellino LE (2001). The role of feedback in the process of health behavior change. Am J Health Behav.

[18] Serlachius A, Schache K, Kieser A, Arroll B, Petrie K, Dalbeth N (2019). Association between user engagement of a mobile health app for gout and improvements in self-care behaviors: randomized controlled trial. JMIR Mhealth Uhealth.

[19] Kanai M, Toda T, Yamamoto K, Akimoto M, Hagiwara Y (2022). A mobile health-based disease management program improves blood pressure in people with multiple lifestyle-related diseases at risk of developing vascular disease　― a retrospective observational study ―. Circ Rep.

[20] Campbell NR, Paccot Burnens M, Whelton PK, Angell SY, Jaffe MG, Cohn J, Espinosa Brito A, Irazola V, Brettler JW, Roccella EJ, Maldonado Figueredo JI, Rosende A, Ordunez P (2022). 2021 World Health Organization guideline on pharmacological treatment of hypertension: policy implications for the region of the Americas. Lancet Reg Health Am.

[21] de Zambotti M, Goldstone A, Claudatos S, Colrain IM, Baker FC (2018). A validation study of Fitbit Charge 2™ compared with polysomnography in adults. Chronobiol Int.

[22] Grundy SM, Stone NJ, Bailey AL, Beam C, Birtcher KK, Blumenthal RS, Braun LT, de Ferranti S, Faiella-Tommasino J, Forman DE, Goldberg R, Heidenreich PA, Hlatky MA, Jones DW, Lloyd-Jones D, Lopez-Pajares N, Ndumele CE, Orringer CE, Peralta CA, Saseen JJ, Smith SC Jr, Sperling L, Virani SS, Yeboah J (2019). 2018 AHA/ACC/AACVPR/AAPA/ABC/ACPM/ADA/AGS/APhA/ASPC/NLA/PCNA Guideline on the management of blood cholesterol: executive summary: a report of the American College of Cardiology/American Heart Association Task Force on Clinical Practice Guidelines. J Am Coll Cardiol.

[23] Agarwal S, LeFevre AE, Lee J, L'Engle K, Mehl G, Sinha C, Labrique A (2016). Guidelines for reporting of health interventions using mobile phones: mobile health (mHealth) evidence reporting and assessment (mERA) checklist. BMJ.

[24] Piette JD, List J, Rana GK, Townsend W, Striplin D, Heisler M (2015). Mobile health devices as tools for worldwide cardiovascular risk reduction and disease management. Circulation.

[25] Masaki K, Tateno H, Nomura A, Muto T, Suzuki S, Satake K, Hida E, Fukunaga K (2020). A randomized controlled trial of a smoking cessation smartphone application with a carbon monoxide checker. NPJ Digit Med.

[26] Kario K, Nomura A, Harada N, Tanigawa T, So R, Nakagawa K, Suzuki S, Okura A, Hida E, Satake K (2020). A multicenter clinical trial to assess the efficacy of the digital therapeutics for essential hypertension: rationale and design of the HERB-DH1 trial. J Clin Hypertens (Greenwich).

[27] Velato L, Cardia G, Romano L, Bartone A, Malizia B, Indolfi C (2022). 976 app-based diagnostic workup of dyslipidemic status in ischemic and not ischemic patients. Eur Heart J Suppl.

[28] Bunting BA, Smith BH, Sutherland SE (2008). The Asheville Project: clinical and economic outcomes of a community-based long-term medication therapy management program for hypertension and dyslipidemia. J Am Pharm Assoc (2003).

[29] Nola KM, Gourley DR, Portner TS, Gourley GK, Solomon DK, Elam M, Regel B (2000). Clinical and humanistic outcomes of a lipid management program in the community pharmacy setting. J Am Pharm Assoc (Wash).

[30] Yogendra J, Yogendra HJ, Ambardekar S, Lele RD, Shetty S, Dave M, Husein N (2004). Beneficial effects of yoga lifestyle on reversibility of ischaemic heart disease: caring heart project of International Board of Yoga. J Assoc Physicians India.

[31] Lv N, Azar KM, Rosas LG, Wulfovich S, Xiao L, Ma J (2017). Behavioral lifestyle interventions for moderate and severe obesity: a systematic review. Prev Med.

[32] Lugones-Sanchez C, Recio-Rodriguez JI, Agudo-Conde C, Repiso-Gento I, G Adalia E, Ramirez-Manent JI, Sanchez-Calavera MA, Rodriguez-Sanchez E, Gomez-Marcos MA, Garcia-Ortiz L (2022). Long-term effectiveness of a smartphone app combined with a smart band on weight loss, physical activity, and caloric intake in a population with overweight and obesity (evident 3 study): randomized controlled trial. J Med Internet Res.

[33] Tong HL, Quiroz JC, Kocaballi AB, Fat SC, Dao KP, Gehringer H, Chow CK, Laranjo L (2021). Personalized mobile technologies for lifestyle behavior change: a systematic review, meta-analysis, and meta-regression. Prev Med.

