# Supplementary material for: Exploring the Feasibility and Initial Impact of an mHealth-Based Disease Management Program for Chronic Ischemic Heart Disease: Formative Study
Source: JMIR Form Res. 2024 Aug 22;8:e56380. doi: 10.2196/56380 (PMC11377902; doi:10.2196/56380)
Supplement: Multimedia Appendix 1 [file formative_v8i1e56380_app1.docx]

Appendix 1 Free comments provided by participants

- "Received appropriate advice from [Expert’s name]."
- "A valuable experience. I used to exercise every day and never thought I could get sick, but due to my family history of diabetes, I eventually did. This program helped change my mindset. Thanks to the mentor's excellent coaching skills, I hope to participate again."
- "Received advice about food and health, and discussed my medical history and future recommendations, which was very helpful. Felt supported and saw meaningful improvements in metrics. Grateful for the opportunities created."
- "Continued motivation is necessary after the program."
- "Endless gratitude to [Expert]. for their warm advice, which helped improve my lifestyle bit by bit. I continue to follow their suggestions on minerals, sugar intake, and lifestyle."
- "[Expert's name] advice was excellent. Lost over 20 kg in six months."
- "It was motivating to see steady progress and results from my efforts."
- "Accurate advice and enjoyable conversations made it possible to engage positively for a long time. Thank you."
- "Thank you for the six months. The person in charge was always cheerful and polite, making the program enjoyable. I will continue to apply what I've learned."
- "Previously interested in health, the advice enhanced and extended my knowledge. However, those without prior knowledge might struggle with self-management."
- "Unable to use Fitbit due to smartphone compatibility issues. Visited major electronics stores, but none carried it. Should increase smartwatch options."
- "Thank you for the good advice. Being able to check my sleep and heart rate timely was very helpful."
- "Enjoyed meetings with the very kind person in charge, looked forward to every session. Thank you."
- "Setting goals for lifestyle improvement and maintaining various daily records is crucial. Continued self-management will be challenging after the program, but I plan to keep using the Mystar app and Fitbit. Thank you for the six months."
- "Encouraged by [Name]'s words and gained knowledge, my health improved significantly. I've also become more interested in my general health and have built up my muscles. I aim to slow aging and extend my healthy years."
- "Regularly scheduled consultations helped maintain motivation for continued improvement."
- "Appreciated the multifaceted assessments and guidance aimed at preparing for the next attempts."
- "For six months, I was deeply grateful for the heightened health awareness. I intend to continue the practices learned. Thank you."
- "Having regular, dedicated time to discuss anything with the healthcare provider was truly beneficial."
- "Fortunate to have met wonderful mentors like [Expert’s name], who provided careful and appropriate support. Realized the importance of mentorship for awareness and persistence. Though the program is over, I plan to continue self-checking and applying what I've learned."
- "Answers to various questions were helpful. I plan to review the readings and continue making an effort. The app was mostly easy to use."
